# Integrating Oral Health Within Kenyan HIV Research & Policy Structure: Stakeholder Analysis

**DOI:** 10.5334/aogh.4150

**Published:** 2024-02-13

**Authors:** Ana Lucia Seminario, Marina Martinez, Immaculate Opondo, Sara Stanley, Matthew Saxton, Arthur M. Kemoli

**Affiliations:** 1 Pediatric Dentistry, School of Dentistry, University of Washington, US; 2 Global Health, School of Public Health, University of Washington, US; 3 School of Dentistry, Universidad Peruana Cayetano Heredia, 6444 74th NE, Seattle, WA 98105, US; 4 Washington Chapter of the American Academy of Pediatrics, US; 5 School of Medicine, Maseno University, KE; 6 UW DeRouen Center for Global Oral Health, School of Dentistry, University of Washington, US; 7 Information School, University of Washington, US; 8 Department of Pediatric Dentistry and Orthodontics, University of Nairobi, KE

**Keywords:** Stakeholder Analysis, Oral Health, HIV, Kenya

## Abstract

**Background:**

Kenya has a remarkably high burden of oral diseases, especially in vulnerable communities like persons with HIV (PWH). In the last few decades, the National AIDS & STI Control Programme has efficiently and successfully provided care and prevention against HIV for people living in Kenya.

**Objective:**

To assess the feasibility of integrating oral health into HIV research and policy structures in Kenya.

**Methods:**

The study took place between November 2021 and April 2022 in the cities of Nairobi, Kisumu, Mombasa, and Eldoret town. Using a semi-structured interview, three remote and 14 in-person sessions were conducted. Participants included individuals with professional experience in HIV and/or oral health such as researchers, potential mentors, institutional administrators, and other grant-funded experts. A qualitative analysis of recordings was performed by nine pretested independent reviewers, all with qualitative data analysis experience. Areas of interest included research, motivation, obstacles, and support. The free coding phase as well as an iterative grouping analysis (MIRO) was used.

**Findings:**

Of the 22 stakeholders interviewed in the study, researchers accounted for the majority (48%) of stakeholders, with the rest composed of practitioners (29%), university administrators (19%), and one public health administrator. University administrators were identified as having the most ability for resource mobilization followed by researchers and practitioners. All participants desired improved health outcomes using an evidence-based approach. The primary motivators were increased networks, collaborations, publications, and bridging the gap between oral health and HIV. While the obstacles to their desires included time and lack of funding, Institutional support through recruitment of qualified personnel, mentors, and mentees was their major desirable support.

**Conclusion:**

Stakeholders were unanimous in supporting integrating oral health within the current research and policy environment to address the gap between oral health and HIV, and to improve health outcomes through evidence-based interventions.

## Background

Oral health research has not yet been prioritized in Kenya, resulting in the prevalence of oral diseases standing at over 50% among persons with HIV, many of which are preventable [1]. Combined, oral diseases are the most prevalent chronic disease in the world [1]. The prevalence of oral diseases is staggering, and more public health research focusing on the issue is critical. The high prevalence of oral diseases is costly in terms of direct financial burden occasioned by managing treatment and its sequelae [2]. Additionally, it has indirect costs due to absenteeism, loss of productivity, and links to other chronic diseases [3]. Further, the disproportionate prevalence and limited access to oral healthcare around the world is a symptom of global health inequity [4].

Unfortunately, many governments, especially those in low- and middle-income countries (LMICs), lack the capacity to conduct quality research on oral disease epidemiology, prevention, and treatment [5]. Thus, this gap presents an opportunity for the development of programs that can reduce those disparities. Effective strategies to alleviate the burden of oral diseases require highly trained health research professionals with in-depth scientific expertise and leadership skills to enable multidisciplinary collaborative teams to develop successful interventions to achieve good oral health. A study analyzing the impact of research training programs in Kenya and Uganda showed that research training programs with a communicative, collaborative network, involving US-based institutions and colleagues, saw stronger proposal writing and grant implementation compared to the lack of university-affiliated training programs [6]. While successful efforts have been made to increase capacity in HIV research and delivery of care, oral healthcare remains inadequate, with limited access in some geographical areas [7].

Stakeholders are vital for the advocacy and support of integration of oral health within HIV research and care in Kenya. These champions are pivotal for building internal research collaboration and for partnering with external organizations for necessary expertise [8]. The purpose of this strategic analysis was to assess feasibility for integration of oral health within current HIV research and policy structures in Kenya. The data collected from interviews informs the creation of an interdisciplinary oral health and HIV research training program in Kenya that will develop the future cadre of local oral health researchers. The specific aim was to identify oral health champions to define organizational structure to support research and training programs in oral health HIV. From this analysis, research training approaches will utilize existing resources and address the HIV oral health training gap that is most suitable to the Kenyan oral health community.

## Methods

### Target population

Our study was approved by the Institutional Review Board and the ethics committee at the University of Washington (STUDY00013617). The ethics committee stated no informed consent was needed as individuals were not identified. Local ethical review committee clearance in Kenya was not logistically feasible with eight institutions [9]. Being in a project funded by the NIH, we opted for one, and the institutions we work with are satisfied with the single ethical clearance. This qualitative study was anonymous, and all participants provided informed consent. Individuals invited to participate included those likely to serve as mentors in our overarching goal of increasing capacity in oral and HIV research in Kenya. To increase diversity, our study population included representatives from Nairobi, Kisumu, Eldoret, and Mombasa. Potential mentors included university professors, clinical researchers, and senior administrators with professional experience in HIV and/or oral health (Table 1).

**Table 1:** Demographic Characteristics of Stakeholders.


CHARACTERISTICS OF STAKEHOLDERS	NUMBER (%)

Title	

- Professor	- 5 (22.7%)

- Dentist	- 5 (22.7%)

- Researcher	- 3 (13.6%)

- Periodontist	- 1 (4.5%)

- Associate Professor	- 1 (4.5%)

- Research Scientist	- 1 (4.5%)

- Pediatrician	- 1 (4.5%)

- [title omitted]	- 5 (22.7%)

Institution	

- University	- 12 (54.5%)

- Clinic	- 8 (36.4%)

- Health Initiative	- 2 (9.1%)

Role	

- Researcher	- 10 (45.5%)

- Practitioner	- 7 (31.8%)

- Administrator	- 5 (22.7%)

Sex	

- Female	- 16 (72.7%)


*Note*. The demographic characteristics of the stakeholders table shows the professional affiliation and sex of stakeholders. The [title omitted] was used to ensure anonymity.

### Data collection and analysis

We conducted 17 interviews involving 22 interview subjects. Three of the interviews were group interviews involving eight subjects, with 2–4 persons in each group interview. These interviews were conducted concurrently, with each respondent sharing their views or contributing to the comments of others. The person speaking first varied for each question.

A semi-structured interview instrument was created to gain an understanding of capacity on oral and HIV research, targeting five relevant areas: support & resources (7 questions), mentors (6 questions), mentees (6 questions), and research & healthcare environment (4 questions). Prior consent was gathered from interviewees, and all interviews were recorded. All identifying information was removed from the transcripts of the recordings. Subjects were assigned a unique code number. All interview materials were kept in a secure location, with only study team access. The recorded materials were transcribed, and each interview ranged between 4–7 pages long, yielding 84 total pages of textual responses for analysis [10].

The researchers employed an inductive approach based on Grounded Theory Methodology (GTM), utilizing constant comparative analysis to derive concepts from data.

The researchers choose to apply an open coding method instead of a priori coding for multiple reasons. The subject area has not been widely examined, and no pre-existing codebook was available. When applying a priori codes, researchers run the danger of overlooking insights from data that do not fit the existing framework. Most of the researchers on the team have deep familiarity with the research and healthcare environments in Kenya and hold opinions on existing challenges from a scholarly perspective; the potential for bias would be high if codes were assigned a priori. The goal of this inquiry was to surface the perspectives of practitioners and administrators in their own voices. Accordingly, open coding was determined to be the most appropriate approach for addressing these challenges as part of a Grounded Theory Method [11]. None of the authors participated in the creation and assigning of codes to the transcripts to avoid introducing bias.

Qualitative analysis was conducted by a group of nine independent reviewers (led by MS) with experience in talking with health care professionals and qualitative data analysis. Each reviewer analyzed one transcript while free coding the text. Transcripts were assigned randomly to reviewers. Reviewers were instructed to capture and utilize the terms or phrases generated by the informants themselves. Prior to beginning the free coding, reviewers took part in a training workshop that used sample responses from multiple transcripts. Coding was conducted at the question response level. The free coding phase of the analysis resulted in 457 unique codes. Since each response could have more than one code, there were many duplicates across transcripts.

Axial coding was performed using a team-based approach. This method has been successfully applied in other health research contexts when the goal is to achieve high intercoder consensus among a single team rather than replicability across different teams of multiple projects [12]. Reviewers were organized into small teams that performed an iterative grouping analysis where each code was typed on a virtual “post-it” note in a collaborative online whiteboard environment (*MIRO*) [13]. Typically, the researchers would conduct this type of exercise in a face-to-face setting, but the need for isolation during pandemic conditions made it challenging to gather physically in a safe manner. It is unclear what effect this may have had on the judgements and decisions or the analysts. One observation is that the practice of zooming in on one segment of the board at a time, rather than continuously scanning the entire “wall” simultaneously resulted in a faster analysis than usual. As notes were shuffled and moved to different categories, the researcher would focus more deeply on each category individually. Through multiple iterations, the reviewers sorted the open codes into groups that appeared to exhibit a “best fit” to the evidence in terms of leaving out the smallest number of uncategorized codes (outliers). Ultimately, 457 open codes were reduced into 16 axial codes organized into four categories: Research, Motivation, Obstacles, and Support. If reviewers were in disagreement, we used the solution that provided a more complete encapsulation of the codes.

An online software app called WordCloud Generator by Monkey Learn was used to create word clouds and diagrams of common words and phrases extracted from the interview transcripts [14, 15]. Word clouds are a useful means to make a large body of text comprehensible, particularly when paired with other qualitative means of analysis. While the constant comparative analysis identified emergent theme categories to enable the creation of an affinity diagram, word clouds, based on frequency counts of open codes, created graphic representations of significant terms and concepts in the actual words of the interview subjects. These word clouds presented an alternative depiction of the data for communicating with audiences less familiar with interpreting coded qualitative data.

## Results

Between November 2021 and April 2022, members of the research team interviewed 22 individuals in 17 sessions. Three sessions were conducted remotely, while 14 were in-person. Researchers accounted for 47.6% of stakeholders interviewed, being the majority in the study. Others interviewed were practitioners (28.6%), university administrators (19.0%), and one public health administrator (4.5%). University administrators were identified as having the most resources and the ability to mobilize them, followed by researchers and practitioners. Our analysis of interview transcripts revealed five emergent themes: Research, Motivation, Obstacles, Support, and Other.

### Research

**Figure 1:**
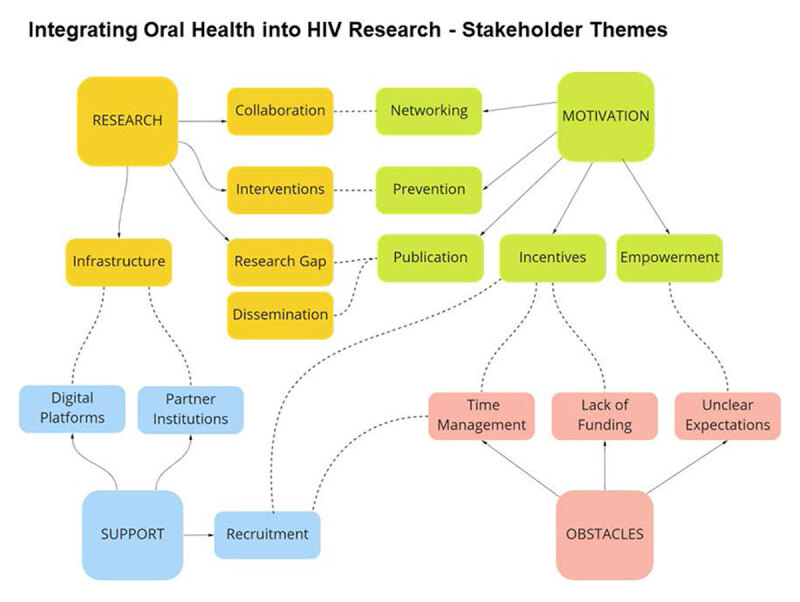
D71 Diagram – Integrating Oral Health into HIV Research – Stakeholder Themes. *Note*. Integrating Oral Health into HIV Research – Stakeholder Themes Diagram shows emergent themes as research, motivation, support, and obstacles resulting from coded data. The solid lines represent sub-themes in reference to a larger theme. The dotted lines represent areas of influence where there is potential for cross-theme impact.

**Figure 2:**
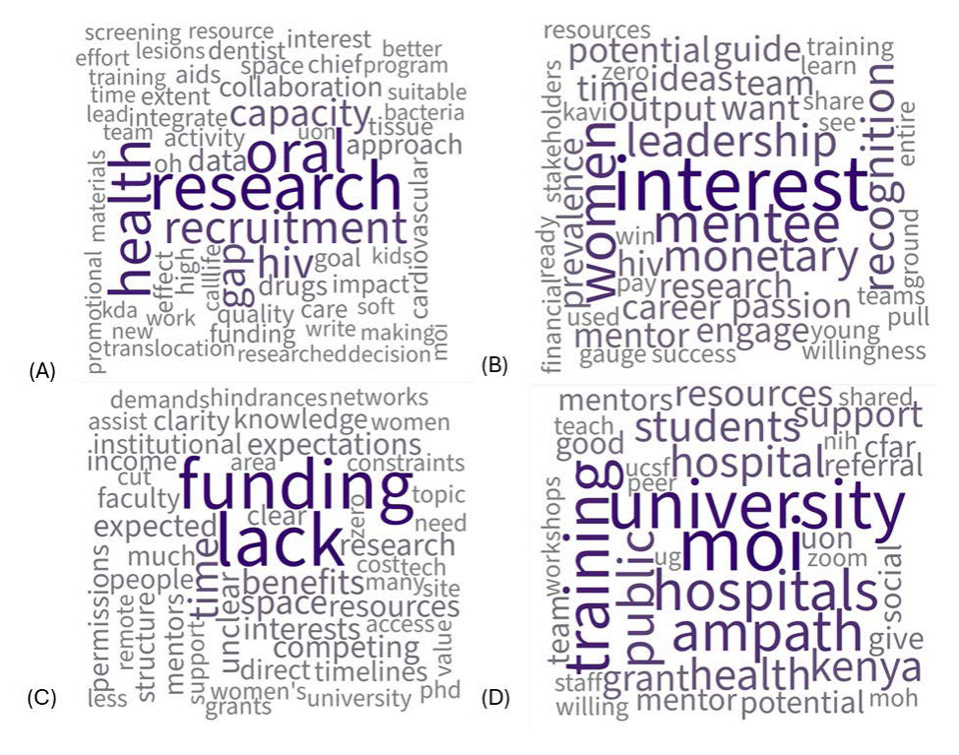
D71 Word Clouds. *Note*. **(A)** The research emergent theme word cloud shows words spoken frequently and related to research in the qualitative interviews we conducted. **(B)** The motivation emergent theme word cloud shows words spoken frequently and related to motivation in the qualitative interviews we conducted. **(C)** The obstacle’s emergent theme word cloud shows words spoken frequently and related to obstacles in the qualitative interviews we conducted. **(D)** The support emergent theme word cloud shows words spoken frequently and related to support in the qualitative interviews we conducted.

Our analysis represented stakeholders across three different categories including practitioners, researchers, and administrators, all uniformly supported the development of a robust research environment to address the gap in understanding the relationship between oral health and HIV. The research emergent theme consisted of sub themes, including collaboration, interventions, research gap, dissemination, and infrastructure. One term appearing in the research word cloud is KAVI, meaning Kenya AIDS Vaccine Initiative (KAVI) (Figure 2A). KAVI was established in 1998 as a research unit within the Department of Medical Microbiology at the University of Nairobi, with an initial mission to conduct basic research in epidemiology of HIV and to carry out HIV/AIDs vaccine trials. Interviewees with KAVI were included as important stakeholders for our project. The research gap in oral health HIV frequently arose in coded data, one participant shared, “In Kenya, oral health has always been left behind. Participation of oral health researchers has not been active.” Another participant expanded, “We have unmet needs of oral health because of a lack of research. We are therefore unsure of the needs because of the lack of data. Our interventions are not evidence based.” Stakeholders were motivated to write publications and contribute to closing the research gap in oral health HIV. The affinity diagram shows the emergent theme research is connected to emergent themes support and motivation (Figure 1). Interestingly, the research theme is not connected to obstacles. Overall, stakeholders were motivated and passionate about research. They expressed interest and highlighted the need to address the research gap of oral health and HIV. Stakeholders were willing to partake in oral HIV research, given a few obstacles were removed (Figure 2A).

### Motivation

The primary motivation for participants to cultivate a research environment examining the intersection of oral health and HIV has three parts. One of these parts was the desire to improve health outcomes by using an evidenced-based approach to interventions. The second one was a desire to extend one’s professional network and collaborate with practitioners and researchers across interdisciplinary lines. The third part was a desire to publish one’s work to share knowledge or advance one’s professional standing. Additionally, stakeholders frequently spoke about their interest in the research gap in oral health and HIV. A large desire exists to improve health outcomes for PWH. Finally, stakeholders want to contribute to evidence-based interventions. One stakeholder expressed the need for research with the following remark, “We have unmet needs of oral health because of a lack of research. We are therefore unsure of the needs because of the lack of data. Our interventions are not evidence-based.” This stakeholder explained the current state of oral health in Kenya, emphasizing a lack of data for evidence-based interventions. Stakeholders recognized the research gap within oral health HIV and strongly wanted to study oral health HIV, write publications, and disseminate their findings to further their careers.

One of the most prominent words on the motivation word cloud was *“mentee”* (Figure 2B). Stakeholders were interested in leading and mentoring the next generation of researchers. Stakeholders were asked several questions regarding what attributes a good mentor and what attributes a good mentee hold. Many participants had a response similar to the following, “A good mentor is one who nests you in their ongoing program in the area of research and ensures lifelong support until one is able to stand on his or her own as a reputable researcher.” In addition to walking with the person they are mentoring, mentors should be able to give their time to regular meetings (Table 2). We also learned about the strong mentoring community established at Moi University, with a participant sharing, “In Moi University, we have mentor training taking place every year and they are linked to the students and these relationships have gone beyond school. Both quotes shared from interviews express strong mentorship bonds sustained throughout their careers.” Stakeholders made it clear; support is needed to sustain the participation of mentors. Though stakeholders used different terminology or had slightly differing ideas of execution, compensation was a theme. Words such as, *“pay”*, *“financial”*, *“resources”*, and *“monetary”*, appeared frequently in the motivation word cloud (Figure 2B). Stakeholders felt lack of compensation was an obstacle to mentorship (Figure 2C). Stakeholders expressed recognition would assist mentor retention. One participant shared, “Recognition is one of the incentives to retain the mentors. Ability to see your mentee succeed in their project.” Most of the factors identified to influence mentor participation were already in place, the motivation to conduct oral health HIV research, opportunities to write publications, network, and access to conduct research and recruit mentees at universities. Despite the influential pieces in place, the emergent theme *motivation* had the second most connections with the *obstacles* emergent theme (Figure 1). Incentives to research oral health and HIV are needed, particularly regarding funding and time management.

**Table 2:** Traits of Mentors and Mentees.


CHARACTERISTICS/TRAITS OF MENTEES	CHARACTERISTICS/TRAITS OF MENTORS

Interest in the research area/Inquisitive Ambitious: can see a path for themselves Self-motivated/Eager Trainable/Willing to learn Accepts criticism Committed/Persistent/FocusedGood listener Able to communicate and share ideas One who can work in a team Protects their time Can assist the mentor in areas where they may be deficient (e.g., technology)	Passion for the research area Qualified health provider or researcher Can identify potential in others Develops potential in others Building capacity in another example Approachable/Patient/Supportive Able to give their time Is available; regular meetings with mentee Ready to walk with a person they are mentoring Does not take over project Realistic about what is or isn’t possible Has the best interest of mentee at heart Willing to sacrifice Nests you in their ongoing program


*Note*. The traits of mentors and mentees table shows common phrases and ideas participants shared about what characteristics and traits constitute a good mentor and mentee. The traits listed are independent characteristics between mentors and mentees.

### Obstacles

Time commitment and lack of funding were frequently cited as the primary obstacles preventing many practitioners, researchers, and university administrators from participating as a mentor. The words *“funding”* and *“lack”* are prominently displayed in the center of the obstacles emergent theme word cloud, indicating the high frequency usage in interviews (Figure 2C). Despite stakeholders’ high motivation, interest in the oral health HIV research gap, and available setting to conduct research at research universities, stakeholders agreed funding was needed for oral health HIV research to be feasible. One stakeholder expressed, “Financial incentive will make people cut down on other hustles and concentrate on mentoring.” Without funding, mentors were unable to prioritize time away from their paying jobs. This stakeholder’s comment also suggests that by funding oral health HIV research, the time management obstacle may also be removed. With stakeholder input, we have narrowed down obstacles to one main barrier: funding.

Outside of the funding obstacle, words such as *“demands”, “institutional expectations”, “timelines”*, and *“constraints”* appeared. These words represented the time management obstacle. Stakeholders spoke of competing priorities and advised that oral health has not been a priority. “With the prevalence of oral diseases standing at over 50% among PWH, efforts targeting oral health should be a priority” [1]. Funding is a large motivation for mentor participation for most stakeholders. Unclear expectations were another theme within the obstacles emergent theme. Mentors need directional support or guidance on clear goals and directions of the research projects and the mentor-mentee relationship. Clear expectations would promote empowerment and increase motivation for both mentors and mentees.

### Support

Several stakeholders identified the support their institutions or agencies could offer in terms of qualified personnel, mentees or mentors, and contributing to recruitment efforts. The word *“university”* was recorded several times in relation to supporting research activity (Figure 2D). The study identified academic institutions as sources of support for research activity and mentee recruitment. A participant stated, “As academic staff, we are expected to do clinical and laboratory services and research and do a publication. If we want to start something, we have the personnel to start.” Academic institutions were well positioned to support oral health HIV research by providing and helping to identify mentors and mentees. At Moi University, a mentor training is held each year and mentors are matched to mentees. With the support of Kenyan universities, personnel were available to research oral health HIV.

One set of interview questions was focused on what makes a good mentor. The findings showed that, according to the stakeholders, a good mentor has the following traits: passion for the research, developing potential in others, realistic, and best interest of the mentee at heart (Table 2). The stakeholders shared a passion for oral health HIV research and desired to mentor, however, many cited a lack of time in their schedules as an obstacle. Tangible incentives like compensation or more time allocated for research would incentivize stakeholders to become mentors (Table 3). Barriers to becoming a mentor included common themes of lack of time, competing priorities, lack of funding, and lack of structure to communicate and sustain mentorship relationships. Based on the affinity diagram, one facet of the *support* emergent theme was recruitment (Figure 1). Incentives are required to conduct recruitment of mentors and mentees due to time management obstacles (Table 3). Although we have identified universities as locations where many potential mentors and mentees are located, funding is needed to allocate time and resources towards recruitment and mentor retention.

**Table 3:** Recruitment of Mentors.


BEST PRACTICES FOR RECRUITING OR INCENTIVIZING MENTORS

**Recruitment** Identify mentors through referral or selection Check professional accomplishments and research output Conduct interviews Have criteria for the task you want them to accomplish

**Incentives** Calling/feel it in the heart Ability to see their mentee success or progress in career Recognition or appreciation for time and commitment Working with good mentees Tangible incentives: compensation, fellowship, or travel funds Allocating more time for research Opportunity for publication Increasing ability to compete for grants Career advancement Opportunity for training in mentorship or other professional development Projects that related to what they are already doing

**Barriers** No time/competing priorities for attention No funding Lack of communication Lack of structured systems or facilitation to sustain mentorship Disagreement between mentor and mentee No direct benefit to the mentor


*Note*. The best practices for recruiting or incentivizing mentors table shows common phrases and ideas participants shared to effectively recruit and retain mentors, along with the barriers to participation mentors face.

In the affinity diagram, support and research are connected by the research infrastructure needed to support digital platforms and partner institutions. One stakeholder informed the interviewer, “It’s about developing platforms and systems that collect information about oral health and HIV.” TABASAMU, a collaboration with the University of Washington and University of Nairobi, needs support to create and operationalize digital platforms with participation from partner institutions.

## Discussion

The overarching aim of this strategic analysis was to explore feasibility for integration of oral health within current HIV research and policy structures in Kenya. The data collected from interviews informs the creation of an interdisciplinary oral health and HIV research training program in Kenya that will develop the future cadre of local oral health researchers. The specific aim was to identify oral health champions to define organizational structure to support research and training programs in oral health HIV. From this analysis, research training approaches will utilize existing resources and address the HIV oral health training gap that is most suitable to the Kenyan oral health community [5].

Our study showed unanimous stakeholder support in developing a research environment to address the gap in understanding the relationship between oral health and HIV [5]. Researchers, faculty, university administrators, and practitioners from a broad range of organizations were interested in furthering oral health HIV research. This finding supports our study purpose; with buy-in from stakeholders across a diverse set of organizations, we may begin integrating an oral health research structure into the existing HIV research and policy structure in Kenya.

We found that stakeholders were particularly motivated to improve health outcomes through evidence-based interventions. This supports our specific aim of identifying oral health champions to support research and training programs. Currently, no existing organizations or the Kenyan government is working to close the oral health HIV research gap, and Kenyans, especially PWH, are suffering [16]. This supports our study purpose of assessing the feasibility of the integration of oral health within existing HIV research and policy in Kenya. The research gap in oral health HIV results in a lack of evidence-based interventions [16]. Our study shows that stakeholders are motivated to conduct oral health HIV research to contribute to evidence-based interventions, and that universities are well positioned to support oral health HIV research and training. Our findings suggest integrating oral health HIV research into existing HIV research and policy in Kenya is feasible [6]. Barriers such as lack of time, competing priorities, lack of funding, and lack of structure, contribute to oral health HIV interventions that are not evidence-based [16]. The integration of oral health research into existing agencies such as National Aids and STI’s Control Programme (NASCOP) will prove mutually beneficial [17]. One of NASCOP’s main goals is to expand HIV testing [17]. Oral health HIV research can build on current HIV research and make an impact towards existing organizational HIV goals [17]. Many dentists see a high volume of PWH; one stakeholder expressed, “In our setting, when you are handling patients, you are most likely encountering patients whose conditions have been complicated by HIV/AIDS.” Since PWH experience increased dental diseases compared to the general population, more PWH visit the dentist to improve their oral health [1]. If oral health HIV research was integrated into HIV research and funded at NASCOP, it would enable studies on oral HIV manifestation. Using oral health HIV manifestation findings, dentists may be trained to identify patients with HIV, and offer HIV testing. Oral health HIV research can increase access to HIV testing, which would build on NASCOP’s goal of expanding HIV testing [17]. A stakeholder explained the value dentistry can bring to existing HIV research and policy, “Dentists by nature of the work are exposed to HIV infected individuals (…) It can provide potential benefits of early screening and detection within the general dental practice (…) Dental clinics give an opportunity for identifying the oral health manifestations [of HIV].” Integrating oral health research into Kenya’s current HIV research and policy will increase accessibility to HIV screening and testing [16]. Oral health HIV evidence-based interventions are a necessary, and missing piece of current Kenyan HIV research and policy [16]. Integrating oral health research into current HIV research and policy will improve health outcomes, inform evidence-based interventions, and help achieve NASCOP’s goal of expanding HIV testing.

Stakeholders are motivated to write manuscript publications and TABASAMU creates a sustainable environment to meet manuscript requirements. The motivation to author manuscripts can be attributed to two main reasons. First, to help lessen the research gap in oral health HIV [5]. Second, to publish one’s work to share knowledge or advance one’s professional standing. TABASAMU would establish a structure where experienced manuscript writers interested in research would mentor the next generation of researchers and teach the skill of manuscript writing and application. TABASAMU training involves teaching the process and structure of writing a manuscript, the necessary fees, and application process. Manuscripts include publication fees ranging in cost from $2,000 to $2,500. By partnering with organizations focused on HIV research, oral health researchers can collaborate with interdisciplinary fields and work together on manuscripts in an organization that currently has funding for HIV research to build on existing HIV research and lessen the oral health HIV research gap [6]. The TABASAMU network and mentorship approach can train stakeholders to write manuscripts and grants to fund research and manuscript publication. TABASAMU creates sustainability through mentorship, connecting organizations, and building on current HIV research. Our findings around manuscript publications support the study’s specific aim of defining structure. Writing manuscripts and applying for grants can be taught and sustained through training programs and mentorship [6]. Stakeholders’ interest in manuscript publications and mentorship reveal a source of support from which structured and supportive programming can be built upon, thereby meeting another specific aim.

Inter-professional collaboration will build on current HIV research and policy by integrating oral health to gather data and understanding the way that HIV presents [6]. Diverse stakeholder groups including university researchers, practitioners, and administrators, were supportive of a research environment to address the gap in understanding the relationship between oral health and HIV [16]. This supports the specific aim of identifying oral health champions to support oral health and HIV research and training. Support is the first step in integrating oral health into existing HIV research and policy [6]. Additionally, stakeholders desired to extend their professional network and collaborate with practitioners and researchers across interdisciplinary lines. Integrating oral health into HIV research and policy would provide stakeholders with the interdisciplinary collaboration they desire while building an oral health HIV database and identifying oral presentation of HIV. TABASAMU requires support to create and operationalize digital platforms with participation from partner institutions. Another specific aim was to identify oral health champions to define structure. In our study, stakeholders reinforced the need for digital platforms to establish sound data storage, encourage collaboration among interdisciplinary stakeholders, and establish program structure. Prioritizing the structure of digital platforms would establish collaboration and integration of oral health into existing HIV research and policy and add to existing HIV research in a collaborative environment [6]. Digital platforms were a strong finding to establish collaboration and integration of oral health into existing HIV research and policy as well to give direction to TABASAMU mentors and mentees.

The main barrier identified in the study was funding, just as another study reported [18]. Based on the results of this study, funding oral health HIV research may also solve the time management obstacle by allowing stakeholders time in their schedules to prioritize oral health HIV research. Despite a desire to decrease the oral health HIV research gap and motivation to publish manuscripts and mentor students, stakeholders agreed funding was needed for oral health research to be feasible. This finding supports the study purpose of understanding feasibility for integration of oral health within current HIV research and policy in Kenya.

In our study, limitations and several strengths exist. Firstly, three interviews were conducted remotely rather than in-person. While the preference was consistency in the setting, given the pandemic and the geographical location of all our interviewees, 86% of in-person data collection was rather successful. Secondly, due to time-limitations, ten participants decided to be interviewed simultaneously. While the results may have been skewed if one person in the interview was swayed when their counterpart shared their opinion first, both individuals were actively vocal in their responses. Thirdly, a small sample size was interviewed. Despite the small sample size, rich data was gathered from the participants, resulting in 457 unique codes, with many duplicates across transcripts. Many stakeholders are from diverse organizations including researchers, practitioners, and university administrators. The heterogeneity of the interviewed stakeholders allowed diverse perspectives of the oral health and HIV research environments in Kenya.

## Conclusion

Our study’s purpose of understanding the feasibility of integration of oral health within current HIV research and policy in Kenya was met with unanimous support to develop a research environment to address the gap in understanding the relationship between oral health and HIV. Additionally, no existing organizations nor the government has worked to close the oral health HIV research gap in Kenya. Our findings will contribute to designing trainings that fit the perceived needs of Kenyan researchers for integrating oral health within current HIV research and policy in Kenya.
